# Assessment of Self-Medication Practices and Knowledge Among Medical Students in Chengalpattu District: A Cross-Sectional Study

**DOI:** 10.7759/cureus.74202

**Published:** 2024-11-22

**Authors:** Nishanth Kumaran Shri P M, Vedapriya Dande Rajasekar, Jasmine M, Surya B.N, Suganthi S, Vijayalakshmi S, Geethanjali Murthy, Kesavan Sundaraboopathy, Arun Kumar R, Harishma Ramesh

**Affiliations:** 1 Community Medicine, Chettinad Hospital and Research Institute, Chettinad Academy of Research and Education, Kelambakkam, IND; 2 Community Medicine, Shri Sathya Sai Medical College and Research Institute (SBV) Sri Balaji Vidyapeeth (Deemed to be University), Ammapettai, IND

**Keywords:** attitude, college students, drug utilization, health literacy, self-medication

## Abstract

Introduction: The practice of self-medication is wherein individuals initiate the use of medications without consulting a healthcare professional. College life is a period marked by academic, social, and personal changes. Due to their greater freedom and the pressure of academic success, students face various health issues. In reaction to perceived health concerns, college students often self-medicate by using over-the-counter or even medicines without medical consultation. Factors such as desire for quick relief and lack of awareness can contribute to self-medication. Mental health concerns, such as stress, anxiety, and depression, may drive students to self-medicate as a coping mechanism.

Aim: The aim of the study is to estimate the prevalence of self-medication among young adults, determine the reasons, extent, and practice of self-medication, and assess self-medication practices, knowledge, and attitudes among medical students.

Materials and methods: A cross-sectional study was used to collect data on the prevalence of self-medication and associated risk factors among college students from a tertiary private medical college in Chengalpattu district between January and March 2024. For the academic year 2023-24, approximately 1,000 students were registered across the first, second, third, and final years of the college. Using a simple random sampling technique, a total of 450 eligible undergraduate medical students were randomly selected. Informed consent was obtained from the participants before the start of the study. All undergraduate students aged 18 and above were included in the study. Students with chronic diseases requiring regular prescription medications and who were not willing to participate were excluded from the study.

Results: The prevalence of self-medication among medical college students was 66.2%. Among the study participants, 45.6% of students had adequate knowledge about self-medication. Around 51% of participants had an attitude that self-medication is acceptable for minor illnesses. In terms of practice, cough syrup was the most frequently used, with 175 (58.7%) of participants reporting its use. Factors such as year of study, gender and self-medication practice had a significant association with knowledge about self-medication.

Conclusion: Self-medication poses a growing risk to individuals' physical health and overall well-being. Hence, it is crucial to encourage responsible medication use and emphasize the importance of consulting healthcare professionals before taking any medication. If left unaddressed, self-medication practices can lead to severe health consequences and undermine the quality of life.

## Introduction

The World Health Organization defines “self-medication as the use of pharmaceutical or medicinal products by the consumer to treat self-recognized disorders or symptoms, the intermittent or continued use of a medication previously prescribed by a physician for chronic or recurring disease or symptom, or the use of medication recommended by lay sources or health workers not entitled to prescribe medicine. Medicines for self-medication are often called “non-prescription” or “over the counter” (OTC) and are available without a doctor’s prescription through pharmacies” [1].

Globally, the prevalence of self-medication varies significantly. Developed countries like Switzerland, Germany, the United States, and Australia report lower prevalence rates of 8%, 11%, 13%, and 11%, respectively. Conversely, self-medication is substantially more common in developing nations such as India, Pakistan, and Nigeria [1]. In India, the prevalence of self-medication is reported at 53% [2].

For over a decade, self-medication has been a widespread practice worldwide, particularly in developing nations. Factors influencing self-medication include age, gender, socioeconomic status, education level, lifestyle, easy access to drugs, patient satisfaction with healthcare providers, drug cost, exposure to advertisements, internet access, family influence, and prior prescriptions or recommendations from advertisements.

Self-medication offers perceived advantages such as reduced healthcare costs and time savings by avoiding doctor visits. However, these perceived benefits often come with significant risks. It can mask the symptoms of chronic illnesses, increasing the chances of adverse effects and delaying proper diagnosis and treatment. Furthermore, self-medication can inadvertently place a heavier burden on healthcare systems, increasing treatment costs for complications arising from its misuse. In India, internet and media promotion, despite being restricted under the Drugs and Magic Remedies Act of 1954, remain significant drivers of self-medication practices [2]. These practices also contribute to treatment failures and, in some cases, aggressive actions against healthcare professionals by dissatisfied patients or their families.

Medical students are particularly prone to self-medication due to their unique challenges, including academic pressures, social challenges, and the transition to adulthood. Stressors such as anxiety, depression, or insomnia may lead them to use OTC drugs or recreational substances as a quick fix. Concerns about confidentiality or social stigma attached to seeking professional help may also contribute to self-medication. Peer influence and a lack of awareness about the risks of improper drug use, such as incorrect dosages or adverse drug interactions, further compound the problem.

Studying self-medication among medical students is crucial, as this group has greater access to healthcare information and represents the future generation of healthcare providers. Their attitudes toward self-medication today could influence their prescribing practices in the future. Therefore, this study aims to assess self-medication practices, knowledge, and attitudes among undergraduate medical students in the Chengalpattu district of Tamil Nadu.

## Materials and methods

A quantitative approach was used with a cross-sectional design to obtain information on the prevalence of self-medication and associated risk factors among college students from a private medical college (Chettinad Hospital and Research Institute, Chettinad Academy of Research and Education) in Chengalpattu district, between January and March 2024. The sample size was calculated based on a 54 % prevalence of self-medication reported in a study by Akande‑Sholabi et al. [3] with a 5% absolute error, a 10% non-response rate, and a 95% confidence interval, the required sample size was determined to be 440, which was rounded off to 450. A list of students studying in the academic year 2023-2024, across the first, second, third, and final years (around 1,000 students) was registered. From this list, 450 students who met the inclusion criteria were randomly selected using simple random technique (lottery method). The eligibility criteria for participant selection were participants above 18 years of age who are not currently taking any medications.

Students above 18 years of age, pursuing an MBBS degree, and both genders were included in the study. Students with chronic diseases such as asthma, hypertension, and diabetes requiring regular prescription medications and students taking medications for known psychiatric illnesses were excluded from the study.

Data collection tools

The data collection process involved the use of a semi-structured questionnaire that had been pre-tested. The distribution of questionnaires to undergraduate medical students in the first, second, third, and fourth years of the MBBS program was conducted after obtaining the necessary permission from the Head of the Institution. The questionnaire contained details about socio-demographic characteristics and questions on knowledge, attitude, and practice of self-medication. A pilot study was done on 10 percent of the sample size to evaluate the content validity of the instrument and find any potential methodological issues. The pilot test was designed to determine the acceptability of the items, the amount of time required to complete the questionnaire, and any potential issues with the data-gathering process. Data from the pilot study were not included in the final results.

All participants who were currently using any method of self-medication or had done so in the past six months were considered to be practicing self-medication [4].

A total of 10 questions were pretested and validated in a pilot study to measure the knowledge related to self-medication. Reliability analysis for the questions was done, yielding a Cronbach’s alpha of 0.88. Each of the 10 dichotomous questions was measured using a yes/no format, where a negative answer corresponded to a score of 0 (No) and a positive answer corresponded to a score of 1 (Yes). The highest possible score that could be achieved was 10, while the minimum score was 0. The median score was calculated to analyze knowledge of self-medication. The median value of the scores was determined to be 6. Those who secured more than 6 were categorized as having adequate knowledge about self-medication, and those who scored ≤6 as having inadequate knowledge. The data collection for this study was conducted using the questionnaire that was developed and tested. The identities of the participants were kept confidential, and they were provided with the contact number and address of the lead investigator. IHEC ethical clearance for this study was obtained from the Institutional Human Ethics Committee on Human Subjects (Approval. No: IHEC-I/2291/23). IBM SPSS Statistics for Windows, Version 23 (Released 2015; IBM Corp., Armonk, New York, United States) was used to carry out the data analysis.

Statistical analysis

Data entry was performed in a Microsoft Excel spreadsheet, followed by analysis conducted with IBM SPSS Statistics for Windows, Version 23 (Released 2015; IBM Corp., Armonk, New York, United States). The descriptive statistics were presented using frequencies and percentages. The chi-square test was done to analyze the association among categorical variables. An unadjusted odds ratio was calculated using bivariate logistic regression techniques, and those variables with a p-value <0.05 were added to a multivariate model applied to determine the adjusted odds ratio and 95% CI was constructed to gauge the estimate.

## Results

A total of 450 students were assessed regarding their practice, attitude and perception regarding self-medication behavior, out of which 53.6% were male students and 46.9% were female students. The mean age of the respondents was 19.26 ± 1.69. The prevalence of self-medication among the study participants was 66.2% (95% Cl: 61.7 - 70.6).

The most common source for self-medication among participants was pharmacy shops (83.9%), followed by family and friends (20.8%) (Table 1). Previous experience (68.5%) and advice from family and friends (57.4%) were the main sources of information (Table 2). Brand selection was primarily influenced by old prescriptions (68.1%), previous experience (36.6%), and peer use (35.6%) (Table 3). The key reasons for self-medication included minor illnesses (53.4%), the need for quick relief (47.7%), and reliance on old prescriptions (31.5%) (Table 4).

**Table 1:** Source for Self-Medication

Sources for self-medication	n (%)
Pharmacy shop	250 (83.9%)
Online shopping	23 (7.7%)
Medical representative	50 (16.8%)
Family & friends	62 (20.8%)

**Table 2:** Source of Information for Self-Medication

Source of information	n (%)
Internet advertisements	51 (17.1%)
Previous prescription	204 (68.5%)
Family & friends	171 (57.4%)
Textbook & magazine	58 (19.5%)

**Table 3:** Criteria for Selection of Particular Brand

Criteria for selection of particular brand	n (%)
Pharmacist	69 (23.2%)
Old prescription	203 (68.1%)
Used by peers	106 (35.6%)
Advertisement	15 (5.0%)
Previous experience	109 (36.6%)

**Table 4:** Reason for Self-Medication

Reasons for Self-Medication	n (%)
Saves time	51 (17.1%)
Quick relief	142 (47.7%)
Have old prescription	94 (31.5%)
Pharmacist advice	35 (11.7%)
Minor illness	159 (53.4%)
Confidence in knowledge	38 (12.8%)

Around 45.6% of the study participants had adequate knowledge about self-medication. Table 5 presents information regarding the knowledge of self-medication, where it has been reported that 310 (68.9%) of people are of the opinion that self-medication could pose negative implications for health. Our study indicates that 44.4% of respondents believe that utilizing OTC drugs without a prescription is a safe practice. Also, 56.7% of students are aware of the potential adverse effects of common self-medication. About 83.3% of respondents say that providing education to students regarding the hazards of self-medication is necessary.

**Table 5:** Knowledge Regarding Self-Medication

S.No	Knowledge regarding self-medication	Yes n (%)	No n (%)
1.	Do you believe self-medication can lead to adverse health effects?	310 (68.9%)	140 (31.1%)
2.	Do you consider it safe to take over-the-counter medications without a doctor’s consultation?	200 (44.4%)	250 (55.6%)
3.	Are you aware of the recommended dosages for common self-medicated drugs?	265 (58.9%)	185 (41.1%)
4.	Do you think self-medication can lead to drug resistance?	330 (73.3%)	120 (26.7%)
5.	Do you believe self-medication can cause drug interactions?	295 (65.6%)	155 (34.4%)
6.	Have you ever used self-medication for a minor illness (e.g., headache, cold)?	280 (62.2%)	170 (37.8%)
7.	Do you think self-medication is a common practice among medical students?	340 (75.6%)	110 (24.4%)
8.	Are you aware of the potential side effects of commonly self-medicated drugs?	255 (56.7%)	195 (43.3%)
9.	Do you consider self-medication a convenient option for minor health issues?	225 (50.0%)	225 (50.0%)
10.	Do you think educational sessions on the risks of self-medication are necessary for students?	375 (83.3%)	75 (16.7%)

Table 6 presents the association between knowledge about self-medication and various sociodemographic variables among study participants. Younger participants (<19 years) had lower knowledge (OR: 1.745, 95% CI: 1.16-2.63, p = 0.008), as did male participants compared to female participants (OR: 1.675, 95% CI: 1.15-2.44, p = 0.007). First-year (OR: 3.279, 95% CI: 1.85-5.80, p < 0.001) and second-year students (OR: 3.678, 95% CI: 2.03-6.65, p < 0.001) were significantly more likely to have lower knowledge compared to fourth-year students. The study found that parental education and occupation significantly influenced participants' knowledge of self-medication. Inadequate knowledge was associated with fathers being graduates (OR: 1.762) or semi-skilled workers (OR: 3.192), and mothers being graduates (OR: 2.113) or skilled workers (OR: 3.140). These findings were statistically significant. Participants with more educated or skilled parents may rely heavily on their parents for advice and guidance on health matters. This dependence could reduce their motivation or need to independently seek knowledge about self-medication practices. Additionally, educated parents might assume the role of primary decision-makers for health, limiting the participants' exposure to critical health literacy skills, leading to inadequate knowledge about self-medication.

**Table 6:** Association Between Knowledge About Self-Medication and Related Variables Among Study Participants * P-value < 0.05: Statistically significant at 95% confidence interval

S.No	Variable	Knowledge (N = 450)		Total (N = 450)	Chi-square	Unadjusted odds ratio (95% CI)	P-value
		Inadequate n (%) n = 245 (54.4%)	Adequate n (%) n = 205 (45.6%)				
1.	Age						
	≤ 19 years	187 (76.3%)	133 (64.9%)	320 (72.1%)	7.121	1.745 (1.16 – 2.63)	0.008*
	> 19 years	58 (23.7%)	72 (35.1%)	130 (28.9%)			
2.	Gender						
	Male	128 (52.2%)	81 (39.5%)	209 (46.4%)	7.275	1.675 (1.15 – 2.44)	0.007*
	Female	117 (47.8%)	124 (60.5%)	241 (53.6%)			
3.	Per capita income as per modified BG prasad classification						
	Class 1 & Class 2	157 (64.1%)	115 (56.1%)	272 (60.4%)	2.976	1.396 (0.95 – 2.04)	0.085
	Class 3 &Class 4	88 (35.9%)	90 (43.9%)	178 (39.6%)			
4.	Year of study						
	First year	108 (44.1%)	70 (34.1%)	178 (39.6%)	26.125	3.279 (1.85 – 5.80)	<0.001*
	Second year	90 (36.7%)	52 (25.4%)	142 (31.6%)		3.678 (2.03 – 6.65)	<0.001*
	Third year	23 (9.4%)	32 (15.6%)	55 (12.2%)		1.527 (0.74 – 3.14)	0.251
	Fourth year	24 (9.8%)	51 (24.9%)	75 (16.7%)		1	1
5.	Father's educational status						
	Schooling	34 (13.9%)	2 8 (13.7%)	62 (13.8%)	7.625	1.370 (0.76 – 2.44)	0.286
	Graduate	125 (31%)	80 (39%)	205 (45.6%)		1.762 (1.17 – 2.64)	0.006*
	Postgraduate	86 (35.1%)	97 (47.3%)	183 (40.7%)		1	1
6.	Father's occupational status						
	Semi-skilled	27 (11%)	7 (3.4%)	34 (7.6%)	12.156	3.192 (1.35 – 7.55)	0.008*
	Skilled	44 (18%)	54 (26.3%)	98 (21.8%)		0.674 (0.42 – 1.06)	0.090
	Professional	174 (71%)	144 (70.2%)	318 (70.7%)		1	1
7.	Mother's educational status						
	Schooling	39 (15.9%)	43 (21%)	82 (18.2%)	14.233	1.037 (0.61 – 1.74)	0.893
	Graduate	122 (49.8%)	66 (32.2%)	188 (41.8%)		2.113 (1.39 – 3.21)	<0.001*
	Postgraduate	84 (34.3%)	96 (46.8%)	180 (40%)		1	1
8.	Mother's occupational status						
	Unemployed	87 (35.5%)	59 (28.8%)	146 (32.4%)	18.630	1.852 (1.19 – 2.86)	0.006*
	Semi-skilled	17 (6.9%)	16 (7.8%)	33 (7.3%)		1.334 (0.63 – 2.79)	0.444
	Skilled	55 (22.4%)	22 (10.7%)	77 (17.1%)		3.140 (1.77 – 5.56)	<0.001*
	Professional	86 (35.1%)	108 (52.7%)	194 (43.1%)		1	1
9.	Self-medication practice						
	Yes	180 (87.4%)	118 (74.7%)	298 (66.2%)	9.708	2.347 (1.12 – 4.8)	<0.001*
	No	26 (12.6%)	40 (25.3%)	152 (33.8%)		1	1

The multiple logistic regression analysis (Table 7) reveals significant associations between several variables and lower knowledge about self-medication. Male gender was associated with an AOR of 1.778 (95% CI: 1.6-2.7, p = 0.009). First-year (AOR: 3.442, 95% CI: 1.6-7.1, p = 0.001) and second-year students (AOR: 4.073, 95% CI: 2.1-8.2, p < 0.001) had higher odds of lower knowledge. Participants with semi-skilled fathers (AOR: 2.993, 95% CI: 1.12-8.1, p = 0.029) and unemployed (AOR: 3.288, 95% CI: 1.8-5.9, p < 0.001) or skilled mothers (AOR: 7.246, 95% CI: 3.4-15.1, p < 0.001) had significantly higher odds of lower knowledge. Practicing self-medication also increased the likelihood of lower knowledge (AOR: 2.144, 95% CI: 1.2-5.1, p = 0.014). Conversely, skilled fathers were associated with lower odds of poor knowledge (AOR: 0.460, 95% CI: 0.24-0.87, p = 0.019).

**Table 7:** Multiple Logistic Regression Analysis to Find Out the Association Between Variables and Knowledge About Self-Medication * P-value < 0.05: Statistically significant at 95% confidence interval

S.No	Variable	P-value	Adjusted odds ratio	95% CI
1.	Gender			
	Male	0.009*	1.778	1.6 – 2.7
	Female	1	1	1
2.	Year of study			
	First year	0.001*	3.442	1.6 – 7.1
	Second year	<0.001*	4.073	2.1 – 8.2
	Fourth year	1	1	1
3.	Father's occupational status			
	Semi-skilled	0.029*	2.993	1.12 – 8.1
	Skilled	0.019*	0.460	0.24 – 0.87
	Professional	1	1	1
4.	Mother's occupational status			
	Unemployed	<0.001*	3.288*	1.8 – 5.9
	Skilled	<0.001*	7.246	3.4 – 15.1
	Professional	1	1	1
5.	Self-medication practice			
	Yes	0.014*	2.144	1.2 – 5.1
	No	1	1	1

Table 8 shows the attitude of study participants towards self-medication; 51.3% of participants consider self-medication acceptable for minor illnesses, while 29.1% find it unacceptable. Only 4.2% plan to continue self-medicating, and 3.3% accept it only in urgent situations. Additionally, 12.0% promote reading medicinal product leaflets, highlighting the need for caution and informed decision-making.

**Table 8:** Attitude Towards Self-Medication

Attitude towards self-medication	Frequency (n= 450)	Percentage %
I will continue with self-medication	19	4.2
Self-medication is acceptable if taken in urgency	15	3.3
Promote reading leaflet of medicinal products	54	12
Self-medication is acceptable if taken for minor illness	231	51.3
Self-medication is unacceptable	131	29.1

**Figure 1:**
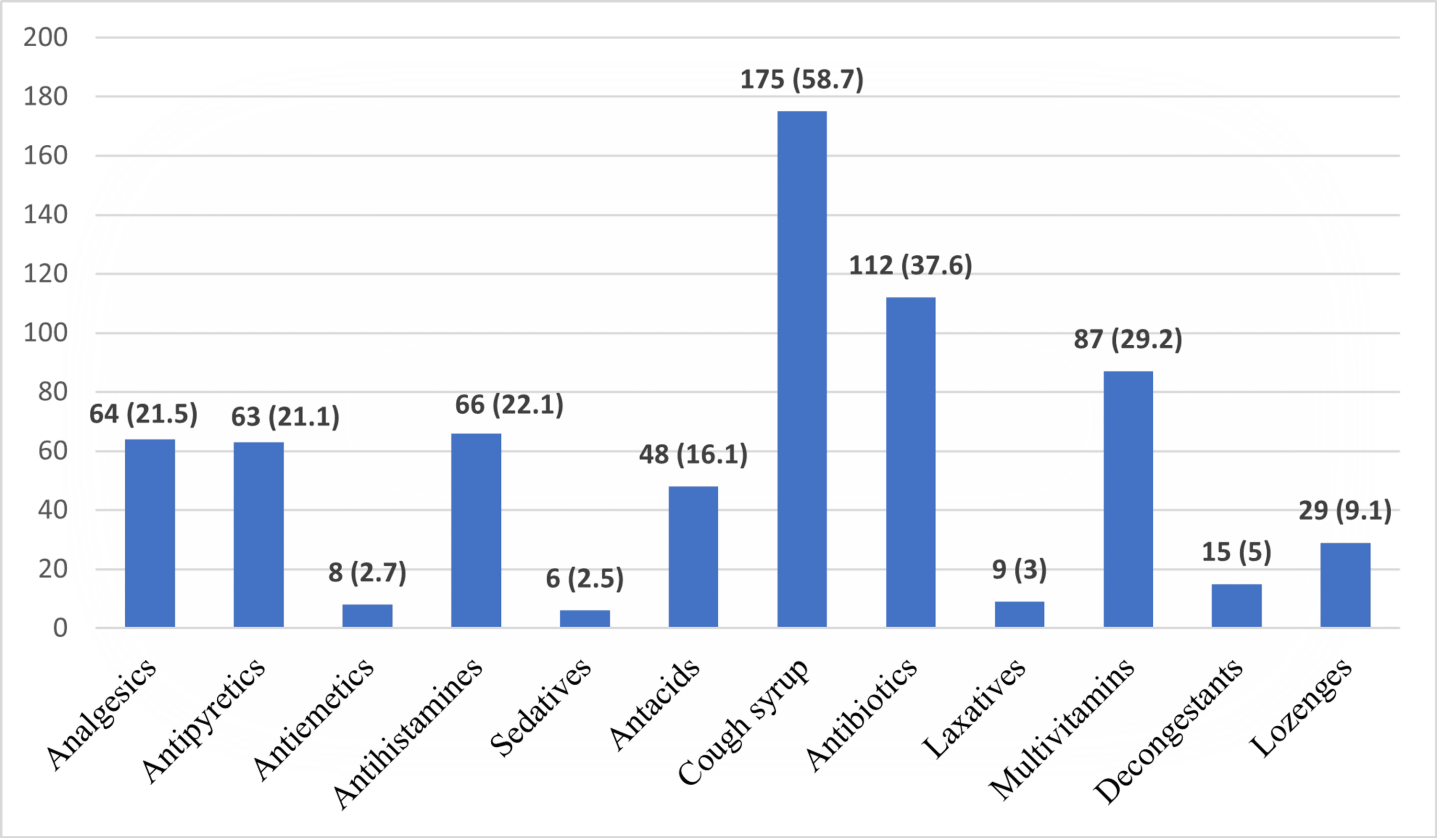
Commonly Used Drugs for Self-Medication (n=298)

**Figure 2:**
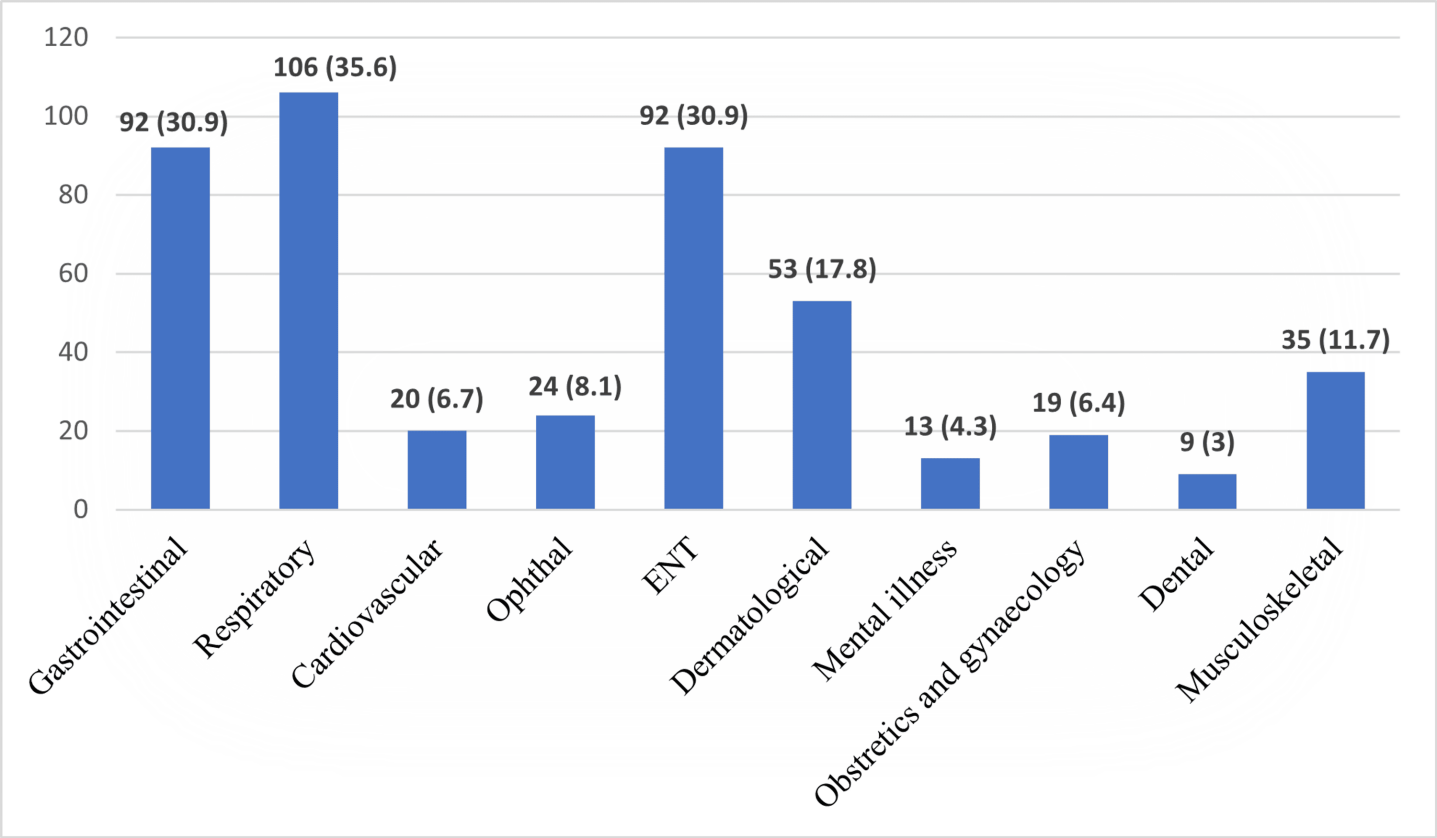
Indicators of Self-Medication (n=298)

Other important findings observed in this study were that cough syrup (58.7%), antibiotics (37.6%), and multivitamins (29.2%) were the most commonly used drugs for self-medication (Figure 1). Respiratory symptoms (35.6%) were the primary indication, followed by gastrointestinal (30.9%) and ENT symptoms (30.9%). Other notable indications included dermatological (17.8%) and musculoskeletal symptoms (11.7%) (Figure 2).

## Discussion

Medical students are at risk of developing a habit of self-diagnosing and self-medicating, which could impact their clinical judgment and potentially affect patient care quality. Self-medication raises concerns due to the risks associated with improper drug use, potential drug interactions, and the development of antibiotic resistance when antibiotics are self-prescribed. As future healthcare providers, medical students' self-medication behaviors may not only affect their health but could also shape their future attitudes and practices regarding patient care.

The prevalence of self-medication among medical students, as observed in the present study (66.2%), along with similar rates found in previous research by Behzadifar et al. [5] (70.1%), Khadka et al. [6] (76.6%), and Banerjee et al. [7] (57%), has significant clinical and public health implications. This highlights the widespread acceptance of self-medication, even among those with formal medical knowledge. Nearly 98% of participants reported that self-medication effortlessly and effectively reduced the symptoms of their illness.

According to the present study, cough syrup and antibiotics emerged as the most commonly used medications. Self-medication with cough syrups poses risks of dependency, overuse, and masking of symptoms that might delay accurate diagnosis of underlying conditions. Additionally, the unsupervised use of antibiotics contributes to a larger public health issue, antibiotic resistance, a global concern that is exacerbated by misuse and overuse of these drugs. This behavior could lead to adverse drug reactions, improper symptom management, and the normalization of unsupervised medication practices that might carry over into their future healthcare careers.

The present study revealed that individuals preferred to obtain their medicines directly from the medical shop, which was comparable to the findings of a prior study in Gujarat [8] and another study in Uganda [9], where most of the participants acquired these medicines via the pharmacy. This trend suggests a gap in access to primary healthcare services or a preference for convenience over formal medical consultation. Pharmacies and medical shops often operate as readily accessible healthcare sources in communities, which may contribute to an increase in self-medication practices.

The study found a high prevalence of self-medication, with many students showing inadequate knowledge about the risks associated with improper use of antibiotics and analgesics. Similar results were observed in studies conducted by Kasulkar et al. [10] among medical students. Many students relied on previous prescriptions or advice from non-professional sources, contributing to inappropriate drug use. These findings reflect that inadequate knowledge about the risks, proper dosages, and safe use of medications often leads to self-medication. Even among medical students, gaps in understanding critical aspects of medication use contribute to the prevalence of inappropriate self-medication practices.

Our study found that 37% of medical students use antibiotics, aligning with the study by Banerjee et al. (31.09%) [7] but lower than the 63.91% reported by Patil et al. [11]. The variation in antibiotic use among medical students across studies emphasizes the need for targeted education and interventions to promote rational antibiotic use.

The finding that 17% of self-medicators in our study reported doing so primarily to save time, as compared to 28.5% in the study by Banerjee et al. [7], highlights an important aspect of self-medication behavior. The desire to save time by avoiding medical consultations may be driven by the perception that immediate relief from symptoms can be achieved more quickly through self-medication. Public health strategies should focus on educating individuals about the dangers of self-medication, emphasizing the importance of consulting healthcare professionals for proper diagnosis and treatment, and promoting awareness of the potential consequences of time-saving approaches to healthcare.

Regarding sources of information, our study found that 68% of self-medicators rely primarily on previous prescriptions, which is in stark contrast to the findings in the Nagpur study by Kasulkar et al. [10] where reading material (52%) was identified as the dominant source. This difference suggests that in our study, individuals may have a more routine or habitual approach to self-medication based on prior medical guidance. On the other hand, Kasulkar’s study reflects a significant reliance on reading materials, which could be indicative of a higher level of health literacy or access to information through various media sources in the Nagpur region.

Our study's finding that 17% of self-medicators use medications primarily to save time, compared to 28.5% in the study by Banerjee et al. [7], highlights regional differences in self-medication behaviors. The lower percentage in our study may reflect better healthcare access, a more cautious population, or cultural differences, suggesting that local factors such as healthcare accessibility and attitudes toward seeking professional care influence self-medication practices.

The findings of our study present a stark contrast to several previous studies on self-medication practices in India, highlighting both regional differences and varying perceptions regarding medication use. For instance, our study suggests a different dynamic, with students relying more on medical shops for sourcing drugs (83.1%) compared to the 65% reported by Kumar et al. [12]. Additionally, Badjger et al. in Mangalore highlighted that 82% of individuals self-medicate for minor ailments [13], a much higher figure than observed in our study. Our data also contradicts the widespread use of analgesics, and antipyretics as reported in studies from Mumbai (63.3% and 68.3%) [14] and Karnataka (50% and 84.9%) [15], respectively, with our rates being significantly lower. The stark differences in utilization rates of these drugs, such as the minimal use of analgesics and antihistamines in our study compared to the study by Shivamurthy et al. (7% and 20.2% respectively) [16], further indicate distinct regional patterns in self-medication. In contrast to the 61.29% reliance on textbooks for self-medication reported in Madhya Pradesh [17], our study found that only 19.5% of students used textbooks as a reference. Finally, the 51.3% acceptance of self-medication in our study is lower than the 62.2% acceptance found in Mangalore [18], suggesting that perceptions of self-medication may vary significantly across regions. These discrepancies highlight the complexity of self-medication practices and emphasize the need for targeted public health interventions to address regional differences.

Our study found 65.6% awareness of drug interactions among students, much higher than the 4.7% reported in an Egyptian study, indicating better understanding in our population [19]. However, awareness of side effects related to self-medication (56.7%) was lower than the 73.9% observed in a study by Raghuprasada et al. [20]. A similar study in the literature [21] also shows that 62.3% of students consider self-medication to be safe, in contrast to our study, which finds that only 44.4% agree with this assessment. These findings emphasize the need for increased education on both drug interactions and side effects, particularly in areas with lower awareness.

Our study found that 73.3% of respondents recognize self-medication as a contributor to drug resistance, similar to the 88.88% reported by Rani et al. in Jammu [22]. This suggests a growing awareness of the risks of self-medication, particularly its role in promoting drug resistance. Both studies emphasize the need for educational campaigns and healthcare interventions to reduce medication misuse.

According to the guidelines established by the World Health Organization for the regulatory assessment of self-medication medicinal products [23], self-medication behaviors are shaped by factors like education, family dynamics, societal norms, legal regulations, drug availability, and advertising. In India, the widespread unregulated sale of drugs, including those that should only be available with a prescription, drives high rates of self-medication. To mitigate risks and maximize benefits, it is essential to implement surveillance systems, encourage cooperation among patients, healthcare providers, and pharmacists, and promote education on safe self-medication practices.

Limitations

The study was conducted among medical college students, which may not represent the self-medication practices and knowledge of the broader population, including students from non-medical backgrounds. Furthermore, recall bias could have influenced the findings, as participants were required to recollect their medication usage over the past six months.

## Conclusions

This study highlights a significant prevalence of self-medication practices among medical students in Chengalpattu District. While self-medication can offer convenience, the lack of professional guidance raises concerns about the potential for adverse effects, misuse of medications, and the emergence of antimicrobial resistance. Targeted interventions, such as educational programs emphasizing the risks of self-medication and promoting responsible medication practices, are essential to ensure safer healthcare behaviors among future medical professionals.

## Appendices

Questionnaire

Socio-demographic Details

1.    Gender

2.    Year of study

3.    Family income per month

4.    Father's educational status

5.    Father's occupational status

6.    Mother's educational status

7.    Mother's occupational status

Knowledge Regarding Self-Medication

1.    Do you believe self-medication can lead to adverse health effects?

2.    Do you consider it safe to take over-the-counter medications without a doctor’s consultation?

3.    Are you aware of the recommended dosages for common self-medicated drugs?

4.    Do you think self-medication can lead to drug resistance?

5.    Do you believe self-medication can cause drug interactions?

6.    Have you ever used self-medication for a minor illness (e.g., headache, cold)?

7.    Do you think self-medication is a common practice among medical students?

8.    Are you aware of the potential side effects of commonly self-medicated drugs?

9.    Do you consider self-medication a convenient option for minor health issues?

10. Do you think educational sessions on the risks of self-medication are necessary for students?

Attitude Towards Self-Medication

1.    I will continue with self-medication

2.    Self-medication is acceptable if taken in urgency

3.    Promote reading leaflet on medicinal products

4.    Self-medication is acceptable if taken for minor illness

5.    Self-medication is unacceptable

Practice of Self-Medication

1.    Have you taken self-medication in the last six months

2.    What was your reason for self-medication

3.    Source of information used for self-medication

4.    Drug commonly taken by you for self-medication

5.    Your selection of a particular brand depends on which of the following choices

6.    Where do you obtain your drug for self-medication

7.    Drugs commonly used by yourself as self-medication

8.    Have you relived after taking medication
